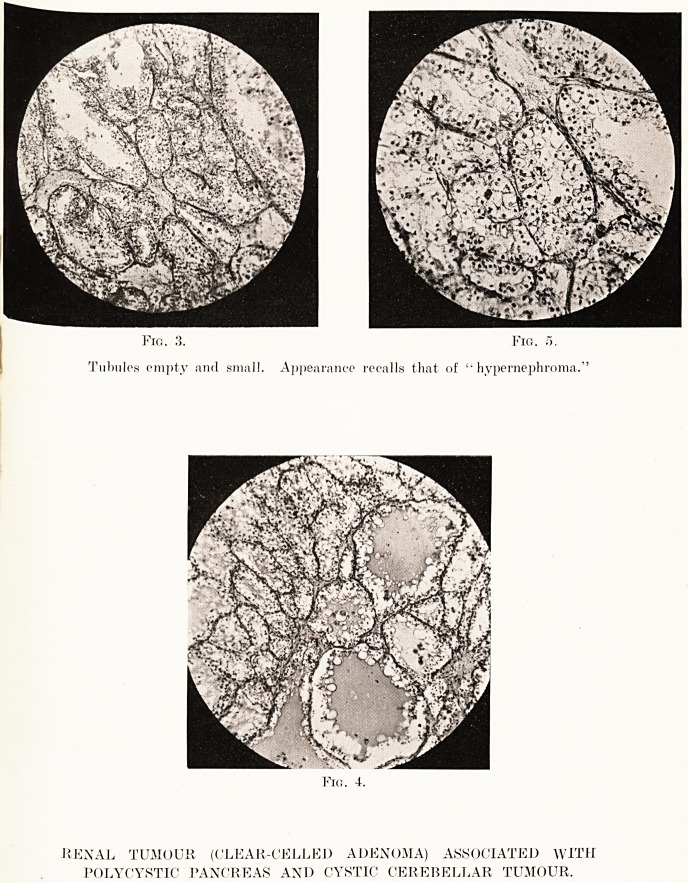# The Association between Angioma of the Cerebellum, Polycystic Pancreas and Renal Adenoma (Lindau's Syndrome)

**Published:** 1929

**Authors:** Geoffrey Hadfield

**Affiliations:** Professor of Pathology in the University of London; Pathologist, Royal Free Hospital, London


PLATE XVIII.

Polycystic Disease of Pancreas. (Four-fifths natural size.)

### THE ASSOCIATION BETWEEN ANGIOMA OF THE CEREBELLUM, POLYCYSTIC PANCREAS AND RENAL ADENOMA (LINDAU'S SYNDROME)

BY

Geoffrey Hadfield, M.D., M.R.C.P.,

*Professor of Pathology in the University of London; Pathologist, Royal Free Hospital, London.*

---

### Introduction.

"Certain maladies that are wholly preventable in the abstract are unavoidable in real deed; for though they may be thought of merely as occupational diseases, unfortunately the occupation is that of existing as a human creature."—Peyton Rous, Linacre Lecture, 1929.

This paper concerns the case of a middle-aged man who died from the pressure effects of a cystic tumour of the cerebellum, probably of congenital origin, who was also found to have a grossly cystic pancreas and an adenomatous tumour of the kidney of appreciable size. At the time of the discovery of these three strange developmental defects in the same individual their association was regarded as nothing more than a bizarre result of chance during the hazards of development, and it was not until enlightened by the distinguished pathologist of the National Hospital, Queen Square, Dr. J. G. Greenfield, that the recorder of this case became aware that these or similar combinations of developmental anomalies have been grouped together as a "syndrome," and that about forty cases have been recorded.

### CASE REPORT.

The patient, a man of 42, was admitted to the General Hospital, Bristol, under the care of Dr. Carey F. Coombs, in the summer of 1927, and died a few weeks later. About a year before death he had a fall on the head followed by a period of unconsciousness and, on recovery from this, by giddiness which persisted and became steadily worse for eight months. Three months before death the giddiness began to be accompanied by tinnitus and was found to be intra-cranial in origin. About this time Mr. Chambers, of the Bristol Royal Infirmary, found the optic discs normal. Two months before death sugar was found in the urine, vertigo was continuous, and he began to suffer from frequent attacks of intense sub-occipital headache with vomiting and bradycardia. The attacks had a sudden onset, lasted a few minutes to half an hour, necessitated morphia for the pain and were accompanied by acute mental confusion. During the attack the pulse would fall to 40 per minute and semi-consciousness with incontinence develop. During two of the attacks lumbar puncture was performed, and from 15 to 20 c.c. of fluid removed under pressure. On each occasion the operation produced striking improvement in the pressure symptoms, consciousness being regained rapidly and the pulse and mental condition becoming normal in a few hours. About this time clinical examination revealed definite sub-occipital tenderness on the right side and brisk knee-jerks, greater in the left than the right, with patellar clonus. The plantar reflex was flexor except on one occasion one month before death, when it became extensor, just preceding and during an attack. There was fine nystagmus greater in the right than the left. The Wassermann reaction was positive (+++) in the blood but negative in the cerebro-spinal fluid.

A moderate and equal degree of bilateral papillœdema was found a month before death, but no other retinal abnormality noticed.

A glucose tolerance test, carried out by Dr. Herbert Rogers, gave the following result :—

| | Urine Glucose. | Blood Glucose. |
|---|---|---|
| Fasting . . . . . . | Nil. | 0·106% |
| ½ hour after glucose | Nil. | 0·193% |
| 1 ,, ,, ,, | 3·3% | 0·137% |
| 1½ ,, ,, ,, | — | 0·068% |
| 2 ,, ,, ,, | 1·2% | 0·056% |

PLATE XIX.

Fig. 1.

Average structure of cystic part. Cysts empty, lined by simple flattened epithelium.

Fig. 2.

Shows development of cysts. To left and above, normal pancreatic acini; to right, groups of dilated and cystic acini.

POLYCYSTIC PANCREAS ASSOCIATED WITH CYSTIC TUMOUR OF CEREBELLUM AND "HYPERNEPHROMA."

PLATE XX.

FIG. 3.

FIG. 4.

Shows transition from dilated acini to microscopic cysts.

FIG. 5.

A field of dilated acini in pre-cystic stage.

POLYCYSTIC PANCREAS ASSOCIATED WITH CYSTIC TUMOUR OF CEREBELLUM AND "HYPERNEPHROMA."

PLATE XXI.

Fig. 6.
Cerebellum. (Half natural size.)

Fig. 7.
Low-power view showing cysts and pre-cystic stage.

Fig. 8.

High-power of 7.

CYSTIC TUMOUR OF CEREBELLUM ASSOCIATED WITH POLYCYSTIC PANCREAS AND "HYPERNEPHROMA."

PLATE XXII.

Fig. 1.

Renal tumour. (Half natural size.)

Fig. 2.

General low-power field. Closely-packed tubules lined by tall, clear cells.

Fig. 2a.

High-power view of 2.

RENAL TUMOUR (CLEAR-CELLED ADENOMA) ASSOCIATED WITH POLYCYSTIC PANCREAS AND CYSTIC CEREBELLAR TUMOUR.

PLATE XXIII.

FIG. 3.

FIG. 5.

Tubules empty and small. Appearance recalls that of "hypernephroma."

FIG. 4.

RENAL TUMOUR (CLEAR-CELLED ADENOMA) ASSOCIATED WITH POLYCYSTIC PANCREAS AND CYSTIC CEREBELLAR TUMOUR.

The inference drawn from this was that the glycosuria was probably pituitary in origin. The patient died a year after the onset of the illness during a period of unconsciousness with Cheyne-Stokes breathing, lumbar puncture on this occasion having no effect on the pressure symptoms.

ABSTRACT OF POST-MORTEM EXAMINATION.

*Brain.*—At least two-thirds of the mass of the right lateral lobe of the cerebellum was replaced by a large, thin-walled multilocular cystic mass filled with clear fluid, pushing the remnant of the lateral lobe downwards and forwards and apparently arising from a broad base of attachment in the middle lobe. The cyst was adherent but not infiltrating, and could be easily peeled off the subjacent cerebellum. After removing the brain the cyst collapsed, and it seemed that the development of the lateral lobe was imperfect and that the cyst had not destroyed it by infiltration or pressure, but was replacing an incompletely developed lateral lobe.

The photograph (Fig. 6) shows the appearance of the tumour after most of its contained fluid had drained away.

Microscopically, the tumour was composed of empty, endothelial-lined spaces varying in size from that of a blood capillary to large, irregularly-shaped cysts up to $\frac{3}{4}$ inch diameter. The intervening tissue was composed of branching neuroglial cells whose processes stained specifically by neuroglial stains. The blood-vessels in the stroma proper were thin-walled and rudimentary but relatively scanty.

*Pancreas.* — This was uniformly and grossly cystic throughout, and its naked-eye appearance immediately recalled that of congenital cystic kidney. The rough preservation of the normal shape and configuration gave the gland the appearance of a caricature of the normal pancreas. The surface cysts varied in size from $\frac{1}{4}$ to $1\frac{1}{4}$ inches in diameter, and on section the cystic change was uniform, even occasional dense, solid-looking areas being finely cystic to the naked eye. The main duct appeared to be a little smaller than normal, but showed no other change. It opened into a normal ampulla.

Microscopically, the walls of the larger cysts had fairly thick walls of mature fibrous tissue lined by simple flattened epithelium. The only normal pancreatic tissue was found in the form of compressed strands of varying thickness lying between the cysts, just as the functioning renal parenchyma lies between the cysts in a congenital cystic kidney. In this tissue cell-islets were numerous and concentrated, but irregular in shape and larger than normal. The impression gained was that the functioning glandular tissue was considerably sub-normal in quantity even when the general enlargement of the organ was taken into account, but that the amount and development of the islet tissue was not greatly below the normal average.

An examination of the dense, solid-looking but finely-cystic masses, such as those seen in the head and tail of the organ in the photograph, showed this to be composed of glandular tissue in which the acini, not arranged in definite lobular form, were all irregularly dilated, and all transitions could be traced from this appearance to the formation of irregular microscopic cysts, and from this to the production of the simple cysts of naked-eye dimensions which studded the surface and cross-section of the gland.

*Kidney.*—Replacing the upper pole of the right kidney was a feebly-encapsulated tumour roughly spherical, about $1\frac{1}{4}$ inches in diameter and projecting only slightly from the surface. It was bright yellow in colour, splashed and dotted with hemorrhagic patches and had a clearly-seen alveolar pattern. Its naked-eye appearances were precisely those of a hypernephroma, except that gross hemorrhage, infiltration and the production of daughter foci were lacking. The suprarenal was normal.

Microscopically, the structure was definitely adenomatous : the gland tubules were lined by a single layer of strikingly clear, empty-looking, low columnar cells with simple, inactive nuclei. The tubules were unequally distended by a homogeneous colloid-like secretion obviously derived from the lining cells, the appearance recalling, apart from the clear cytoplasm of the lining cells, that of a thyroid gland in the stage of colloid storage. The larger individual tubules had exactly the structure of the small congenital cysts so often found in otherwise normal kidneys. The appearances clearly warranted a diagnosis of clear-celled adenoma of the kidney, in all probability of congenital origin, and of a similar nature to the common small solitary cysts often found in otherwise normal kidneys.

### Commentary on Case Report.

The dramatic amelioration of the symptoms of increased intra-cranial pressure following lumbar puncture made a striking impression on all who saw the patient during life, and would appear to be due to relief of pressure due to rapid recurrent accumulation of fluid in the cystic cerebellar tumour. Why the cells composing what appears to be a congenital tumour should produce fluid at this rate for the last eight months of the patient's life is not clear, but it was probably due to some accident to its vascular supply.

The urgency of the pressure symptoms was probably due to the anatomical situation of the cyst. The abnormality in carbohydrate metabolism as revealed by the estimation of glucose tolerance was confidently taken as being part of the clinical picture of increased intra-cranial pressure and of pituitary origin, until the widespread cystic change in the pancreas was found, when it was justifiably concluded that the cystic pancreas must have had some share in the production of the glycosuria. The histological evidence, however, supports the clinical view, as the amount of islet tissue in the pancreas seemed adequate.

The clinically silent renal tumour is of interest in that it supports the modern contention that some, if not all, the Grawitzian tumours of the kidney are actually renal adenoma or adeno-carcinoma, and do not originate in adrenal rests. This yellow hypernephroma-like mass had no connection with the suprarenal, its tubules, allowing for the accident of retention of secretion, were comparable in structure with renal tubules segregated from their glomeruli, and its cells were loaded with yellow lipoid, giving the empty appearance in paraffin sections so typical of the classical Grawitz tumour.

The fascination of the case, however, lay in the association in the same patient of three apparently unrelated pathological conditions, all of which were very probably of congenital origin, and one of which, the cystic pancreas, was excessively rare.

The following discussion is a résumé of the main facts known about this strange association.

### DISCUSSION.

The case described falls into line with a group of cases discussed by Lindau of Lund [1] three years ago. Lindau's investigations were initiated by a careful examination of two cystic tumours of the cerebellum, which encouraged him to ransack many European museums for similar specimens. He discovered that cystic cerebellar tumours are not infrequently associated with angioma of the retina, of the medulla, spinal cord or cerebrum, sometimes with a polycystic pancreas and now and then with renal "hyper-nephromata" or renal cysts. All these conditions may be found in the same patient.

His figures are :—

Of all cerebellar tumours found 10 per cent. were cystic. 275 cases of cystic cerebellar tumour were collected, of which 235 were simple cysts of unknown pathology or gliomatous cysts; 40 were capillary angiomata, and of these 15 showed angiomata in the retina, medulla, spinal cord or cerebrum, 8 had a polycystic pancreas, 10 had cysts of the kidney, and 8 had hyper-nephromata.

Other cases have been described by Schuback [3] and Schubert. [4]

Cushing and Bailey [6] find that tumours of angiomatous structure comprise about 2 per cent.

of all intracranial tumours. Of 29 tumours of this type, 16 were cerebellar angioblastomas. They fully confirm Lindau's findings.

The relation to angiomatosis of the retina is interesting, for the presence of this easily-observed sign during life may give the clue to the presence and nature of an intra-cranial growth which has a relatively good surgical prognosis.

In one case investigated by Lindau[1] eight years before death an eye had been excised for "arterio-venous aneurysm" of the retina. Re-examination showed this to be actually a retinal capillary angioma. Harvey Cushing and Bailey [2] report a case of angioma of the cerebellum operated on at Cushing's clinic in which retinal angiomatosis was found on re-examination, having been missed at the time of operation. The father and aunt of this patient had died of "ruptured cystic sarcoma of the brain."

Lindau finds that 20 per cent. of all cases of retinal angiomatosis collected have had intra-cranial complications. [1] The disease, also known as Hippel's disease, is often familial, is usually peripheral, but definite changes at the disc are described. Secondary changes obscuring the main lesion are frequent.

Parkes Weber [5] in a recent article draws attention to the occasional association of extensive hemangiomatous nævus of the skin with a similar nævoid condition of the cerebral meninges on the same side. In some of his cases there was also a congenital glaucoma. There seems to be no intermediate group between the Lindau syndrome and the cases described and collected by Parkes Weber, but the multiple congenital malformations have some similarity.

REFERENCES.

[1] Lindau, *Acta Path. Scand.*, Copenhagen, 1926, Supp. I.

[2] Cushing and Bailey, *Arch. of Ophthal.*, New York, 1928, lvii., 447.

[3] Schuback, *Zeitschr. f. d. ges Neurol. u. Psychiat.*, Berlin, 1927, cx. 359–371.

[4] Schubert, *Klin. Woch.*, Berlin, 1927, vi. 821.

[5] Parkes Weber, *Proc. Roy. Soc. Med.*, Sect. of Neurol., 1929, Vol xxii., No. 4, pp. 431–442.

[6] Cushing and Bailey, *Tumours Arising from the Blood Vessels of the Brain*, London, 1928.

## Figures and Tables

**Figure f1:**
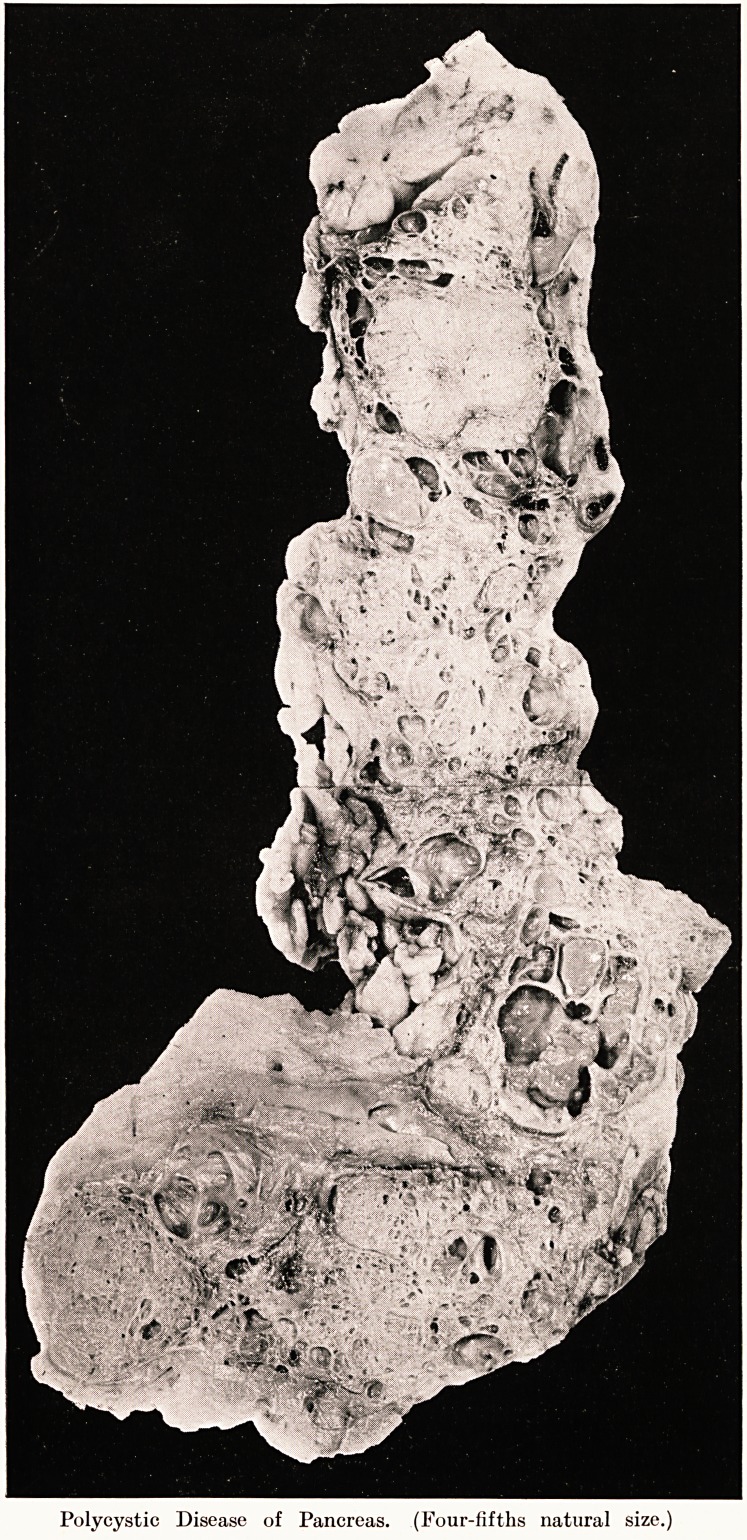


**Fig. 1. Fig. 2. f2:**
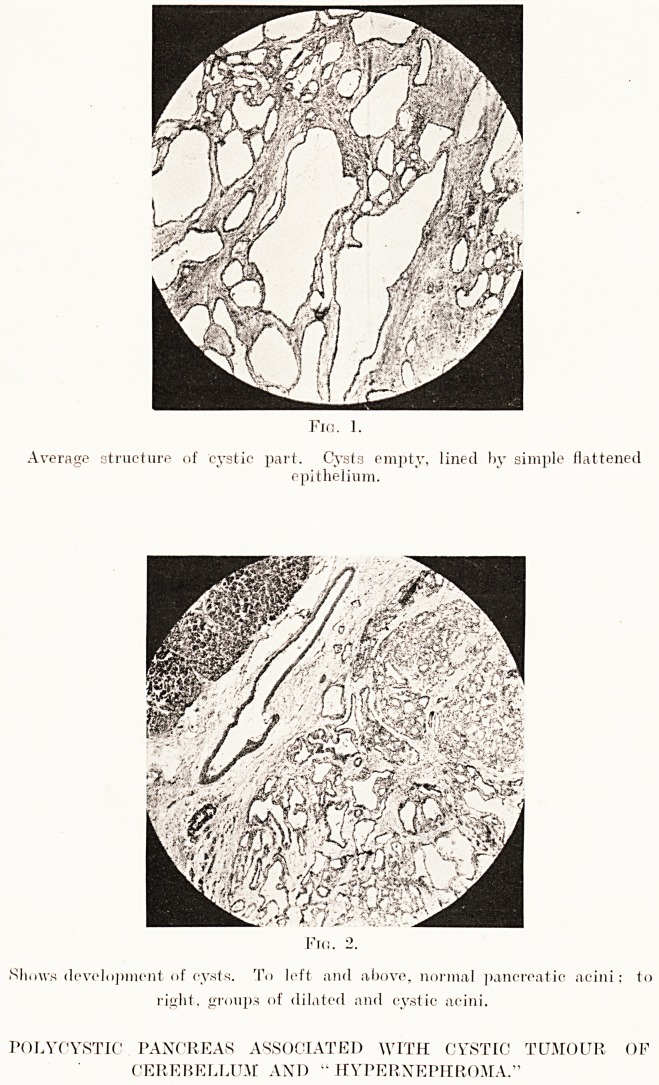


**Fig. 3. Fig. 4. Fig. 5. f3:**
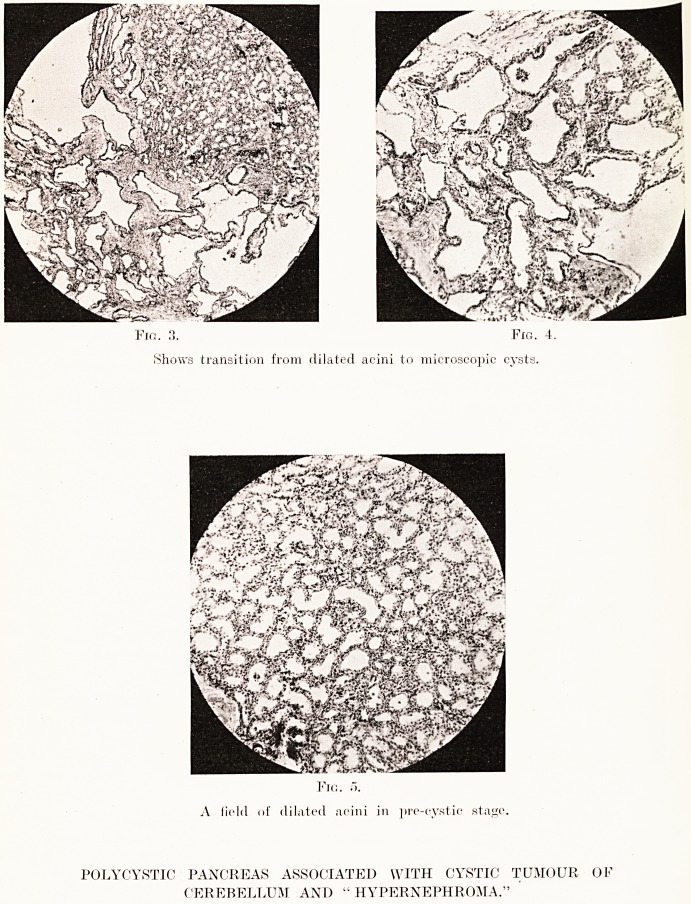


**Fig. 6. Fig. 7. Fig. 8. f4:**
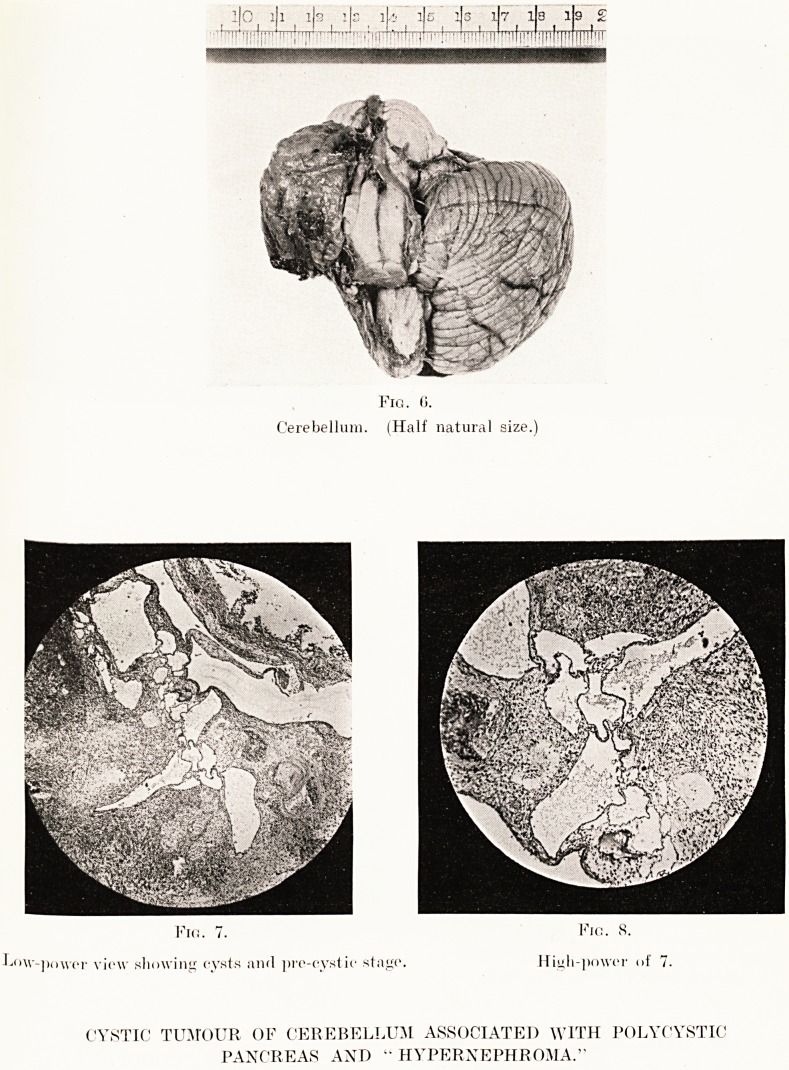


**Fig. 1. Fig. 2. Fig. 2a. f5:**
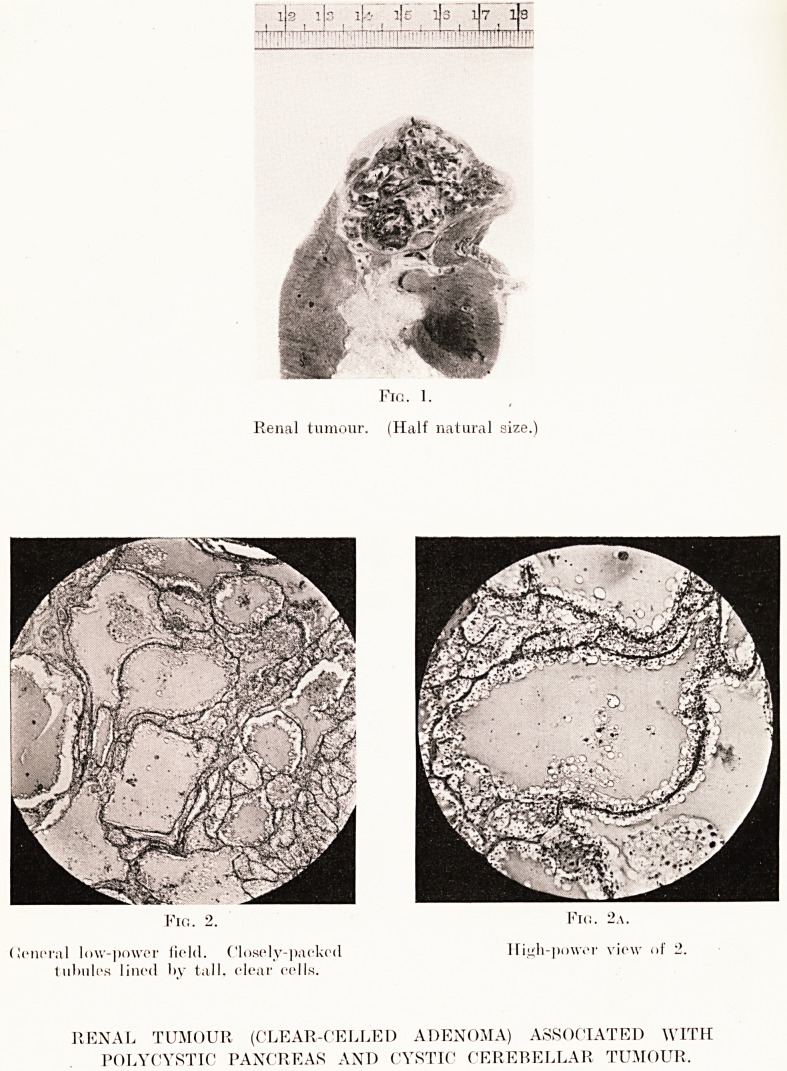


**Fig. 3. Fig. 4. Fig. 5. f6:**